# Debulking Surgery for Elephantiasis Nostras With Large Ectatic Podoplanin-Negative Lymphatic Vessels in Patients With Lipo-Lymphedema

**Published:** 2014-02-28

**Authors:** Uwe Wollina, Birgit Heinig, Jaqueline Schönlebe, Andreas Nowak

**Affiliations:** ^a^Department of Dermatology and Allergology, Academic Teaching Hospital Dresden-Friedrichstadt, Dresden, Germany; ^b^Department of Physical Therapy and Rehabilitative Medicine, Academic Teaching Hospital Dresden-Friedrichstadt, Dresden, Germany; ^c^Institute of Pathology “Georg Schmorl”, Academic Teaching Hospital Dresden-Friedrichstadt, Dresden, Germany; ^d^Department of Anesthesiology and Intensive Medical Care, Emergency Medicine and Pain Management, Academic Teaching Hospital Dresden-Friedrichstadt, Dresden, Germany

**Keywords:** lipo-lymphedema, decongestive lymphatic therapy, tumescent liposuction, adjuvant debulking surgery, elephantiasis nostras

## Abstract

**Objective:** Elephantiasis nostras is a rare complication in advanced lipo-lymphedema. While lipedema can be treated by liposuction and lymphedema by decongestive lymphatic therapy, elephantiasis nostras may need debulking surgery. **Methods:** We present 2 cases of advanced lipo-lymphedema complicated by elephantiasis nostras. After tumescent microcannular laser-assisted liposuction both patients underwent a debulking surgery with a modification of Auchincloss-Kim's technique. Histologic examination of the tissue specimen was performed. **Results:** The surgical treatment was well tolerated and primary healing was uneventful. After primary wound healing and ambulation of the patients, a delayed ulceration with lymphorrhea developed. It was treated by surgical necrectomy and vacuum-assisted closure leading to complete healing. Mobility of the leg was much improved. Histologic examination revealed massive ectatic lymphatic vessels nonreactive for podoplanin. **Conclusions:** Debulking surgery can be an adjuvant technique for elephantiasis nostras in advanced lipo-lymphedema. Although delayed postoperative wound healing problems were observed, necrectomy and vacuum-assisted closure achieved a complete healing. Histologic data suggest that the ectatic lymphatic vessels in these patients resemble finding in podoplanin knockout mice. The findings would explain the limitations of decongestive lymphatic therapy and tumescent liposuction in such patients and their predisposition to relapsing erysipelas.

Lymphedema is a chronic, progressing, debilitating disorder either due to primary malformation of lymphatic vessels or due to trauma, malignancies, filariasis, relapsing erysipelas, surgery, and/or radiotherapy leading to lymphatic insufficiency.^1^ The backbone of treatment is decongestive lymphatic therapy (DLT) that consists of manual lymphatic drainage, compression, mobilization, and hygienic and skin care measures.^2^^-^5

Lipedema is a disorder of abnormal growth and distribution of adipose tissue mostly of the proximal extremities. Lipedema is associated with bruising and pain. The disease affects females during or after puberty. Although an increased body mass index can be observed, the disease is different from obesity. They differ not only by the distribution of adipose tissue (peripheral vs central). In addition, lipedema does not respond to diets, drugs, and physical activities. It can lead to severe impairment of joint mobility (in particular of the knees) and it involves the risk of developing metabolic syndrome.^6^^,^^7^ The only available treatment to improve the long-term outcome is microcannular liposuction in tumescent anesthesia.^8^^-^12

Although lipedema primarily is not associated with disturbances of lymphatic flow, advanced cases may develop a secondary lymphedema resulting in an overlap disease known as lipo-lymphedema.^6^

Elephantiasis characterizes a disfiguring end-stage of lymphatic disease (stage III according to the International Lymphatic Society) with profound but circumscribed changes in soft tissue. Tissue fibrosis, fat deposition, hyperpigmentation, cutaneous acanthosis, and papillomatosis are present. The skin develops a cobble-stone surface pattern. The disease is slowly progressive. These patients possess an increased risk for soft tissue infections that can aggravate the condition. Debulking surgery is an option for selected cases.^1^^,^^13^

We report on 2 female patients with elephantiasis nostras developing secondary on lipo-lymphedema of the legs, who were treated by microcannular liposuction and debulking surgery.

## PATIENTS

### Patient 1

A 76-year-old woman presented with a long-standing lipo-lymphedema that progressed over the last 25 years. She suffered from arterial hypertension, stable angina pectoris, chronic venous insufficiency of the legs and osteoarthritis. Therefore, she underwent a total knee endoprosthesis on the right side several years ago.

She suffered from pain in her legs and reported bruising after minor trauma. Decongestive lymphatic therapy has been performed on outpatient base and once in a specialized lymphedema clinic. Twenty-five years of DLT could not prevent her increasing immobility and chronic pain.

**Figure 1:**
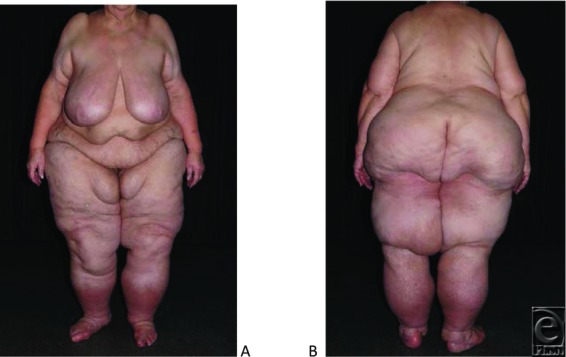
Patient 1 with advanced lipo-lymphedema before liposuction. (*a*) Frontal view; (*b*) posterior view.

On examination, we found a female with typical disfiguring hypertrophy of subcutaneous adipose tissue of the extremities, more pronounced in the proximal parts. She had a positive Stemmer sign on her toes. Her body mass index was 61.6. She was scored grade III lipedema of her legs and grade II-III lipedema of her upper arms (Fig 1).^1^^,^^6^

In a first step, we performed 980 nm–diode laser-assisted microcannular liposuction with tumescent anesthesia. For tumescence, the following solution was used: 1 mL 1:1000 diluted adrenalin, 5 mL prilocaine, and 1000 mL Ringer's solution. The 0.1% prilocaine tumescent solution is less cardiotoxic than lidocaine but needs postsurgical monitoring of met-hemoglobulin. Toluidine blue 20% solution was given intravenously as an antidote.^8^ During 3 sessions, 6400 mL adipose tissue were removed.

**Figure 2:**
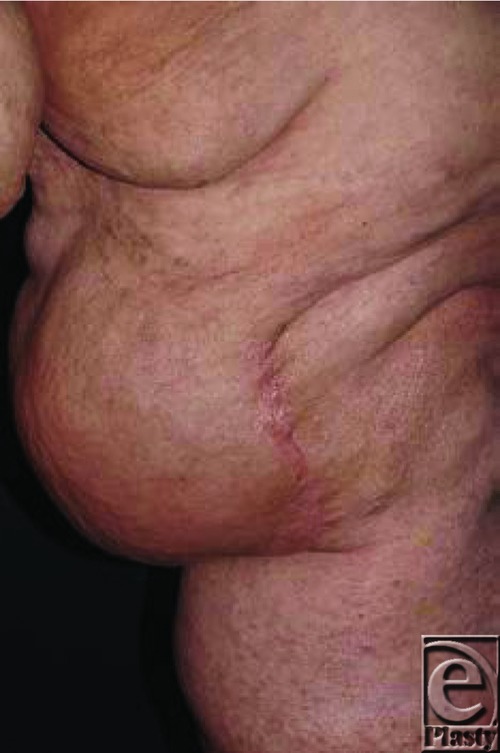
Patient 1, elephantiasis nostras of the distal inner left thigh.

On her left medial knee, circumscribed elephantiasis did not respond to liposuction and DLT (Fig 2). Because of a persistent pain at the left medial knee region, we decided to perform a dermatosurgical resection. By preoperative scoring according to the American Society of Anesthesiology (ASA) Physical Health Score, she was class II: patients who have severe systemic disease that is not incapacitating.^14^

**Figure 3:**
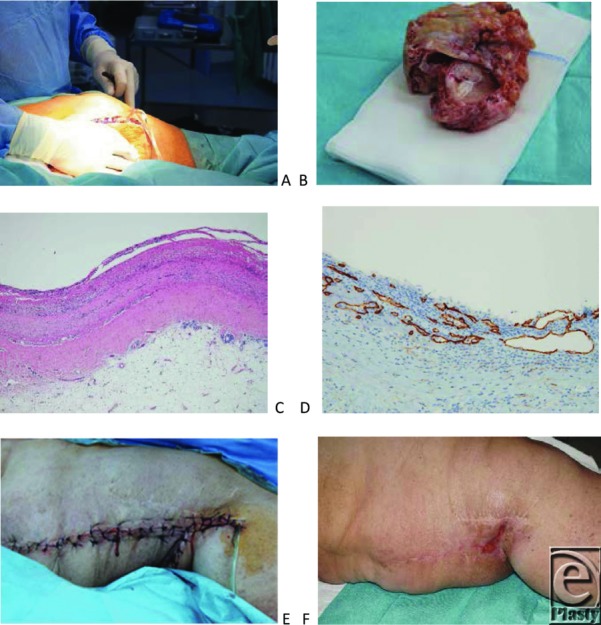
Debulking surgery. (*a*) Intraoperative view on the large pseudocyst with lymphatic fluid emptying. (*b*) Resection specimen of the pseudocyst. (*c*) Wall of the pseudocyst (hematoxylin-eosin, x4). (*d*) Podoplanin-staining of lymphatics in the wall of the pseudocyst, whereas the inner surface remained unstained. (*e*) Operation situs after debulking surgery. (*f*) Near complete secondary wound healing after vacuum-assisted closure therapy. Markedly improved inner thigh contour.

A modified procedure according to Kim et al^13^ with perioperative prophylactic antibiotics was employed. A vertical fish-shaped incision of the skin above the elephantiasis region was made. Then anterior and posterior flaps of 1- to 2-cm thickness were prepared. Subcutaneous tissue was excised down to the aponeurotic plane. Aponeurosis was thinned but not removed. An area of 15 × 10 cm^2^ was dermato-fibro-lipectomized this way. During surgery huge ectatic and fibrotic “vessels” embedded in fibrotic and large-lobulated adipose tissue became evident. A pseudocystic structure of 10 × 5.8 cm^2^ was surgically removed. Intraoperatively, the pseudocyst emptied about 200 mL of lymphatic and serous fluid (Fig 3).

Thereafter, a Redon drainage was placed beneath the flaps. The wound was closed by 2-layered sutures of polyglactin (Vicryl 1, Ethicon) or poly p dioxanone (PDS 0; Ethicon) subcutaneously followed by polyglycolic acid (Prolene 3/0, Ethicon) skin sutures. Compression bandages were taped around the leg. Later, 2-layered compression bandages were used. For ambulatory care compression, garments with stockings and trousers were prescribed. So far, the healing course was uncomplicated.

The histological preparation revealed fibrotic tissue, ectatic blood, and lymphatic vessels. A large pseudocyst was seen without any endothelial component but podoplanin positive lymphatic spaces within its thick wall (Fig 3c). Ectatic vessels were podoplanin negative on the inner surface.

**Figure 4:**
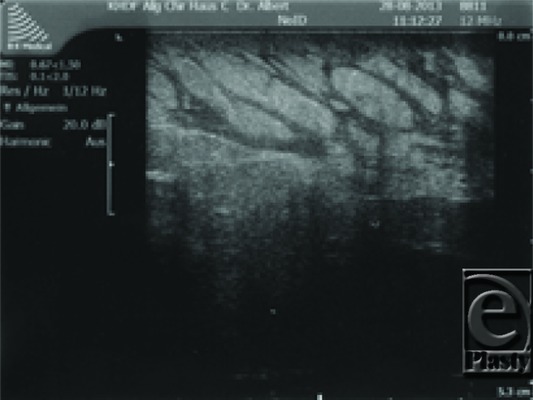
Postoperative sonographic control with large lymphatics but without pseudocysts.

The Redon drainage could be removed after 11 days. Postoperatively, sonographic controls were unremarkable (Fig 4).

One week later, she presented with acute erysipelas of the left leg and lymphorrhea. We initiated systemic treatment with cefuroxime 1.5 g every 8 hours intravenously. After complete remission of the soft tissue infection and wound debridement, vacuum-assisted closure (KCI International) was applied. We started with a black sponge but continued later on with white sponges after partial remission of the lymphorrhea. We achieved an excellent granulation and reepithelialization from all wound margins. The wound area became continuously reduced, and lymphorrhea was completely stopped after 3 weeks (Fig 3f). Complete reepithelialization was seen 1 week later.

The patient experienced an improved mobilization, the leg became painless and the leg shape was normalized. The diameter of the left leg (above the knee) could be reduced from 93 cm (before liposuction) cm to 62 cm (after debulking surgery).

### Patient 2

**Figure 5:**
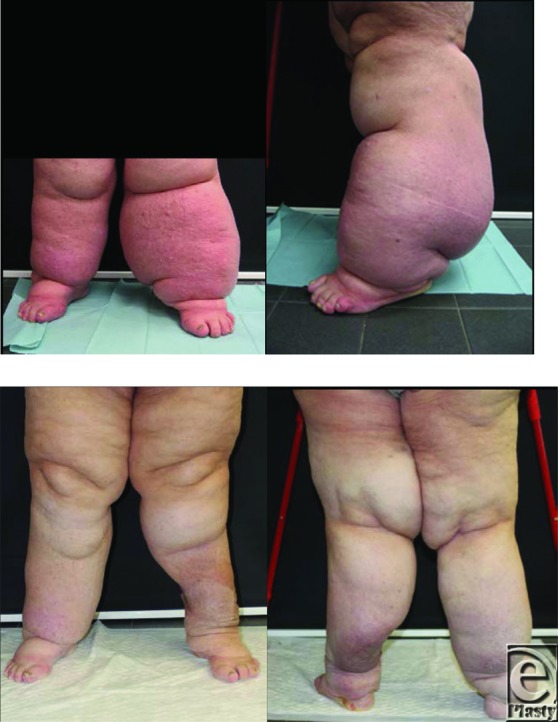
Patient 2. Massive elephantiasis nostras of the left lower leg. Upper row: Before liposuction (*a*, *b*). Lower row: After liposuction of the left lower leg and debulking surgery (*c*, *d*). Mobility was markedly improved.

A 74-year-old woman with an advanced lipo-lymphedema of her legs was admitted to our hospital. She suffered from several comorbidities including diabetes mellitus type IIb, hyperlipidemia, essential hypertension, and postpolio syndrome with paretic symptoms on her left body side. She was unable to climb stairs anymore (Fig 5).

She had got sporadic DLT but without sufficient compression therapy for more than 30 years. The disfigurement especially of the left lower leg was progressive over time. The circumference reached double the size of the contralateral lower leg. During the last years, she suffered from recurrent erysipelas.

On examination, we observed a lymphedema grade III of the legs with elephantiasis of the middle part of her lower left leg. The skin was thickened with a verrucous and papillomatous cobble-stone surface pattern. Stemmer sign was positive on her toes. The lipedema was scored grade III on legs and upper arms. Nevertheless, the major suffering came from her left lower leg. Her body mass index was 62.9.

After conservative treatment with DLT including tailored compression garments, we continued with diode laser-assisted liposuction with tumescent anesthesia for the lower left leg. In 2 sessions, 3100 mL fat tissue could be removed. Healing was uneventful.

**Figure 6:**
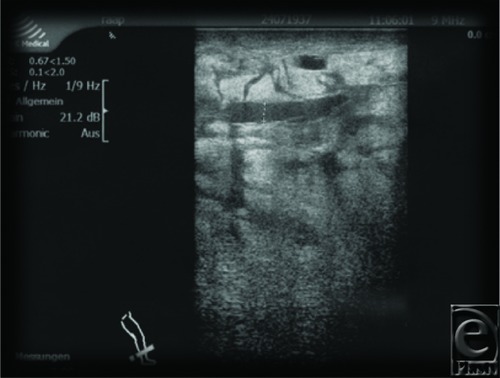
Patient 2. Preoperative sonographic control with massive enlarged lymphatics.

Since the elephantiasis was progressive despite intensified DLT, we decided to do debulking surgery in general anesthesia. Her ASA score was class II.^14^ Preoperative sonography of elephantiasis demonstrated disseminated fat vacuoles with a circumscribed pool of fluid in a depth of 4 to 7 mm (Fig 6).

**Figure 7:**
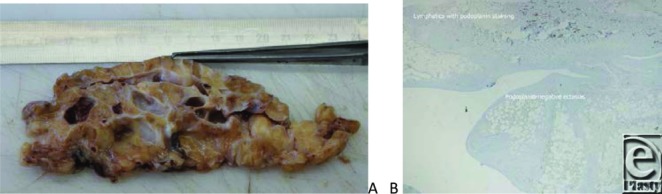
A slice of the surgical specimen after debulking demonstrating severe fibrosis. (*a*) Markedly enlarged lymphatic vessels and phlebectasias. (*b*) Immunostaining for podoplanin: positive reaction in smaller lymphatics but loss of podoplanin reactivity in ectatic spaces (x4).

By debulking surgery, a 50 × 25 × 10 cm^3^ portion was dermato-fibro-lipectomized (Fig 7).

Fibrosis, large-lobulated adipose tissue with ectatic blood and lymphatic vessels, was seen. Histologic investigations demonstrated multiple interconnected and slit-like phlebectasias and podoplanin-negative pseudocystic spaces without any endothelial layer (Fig 7b).

With the surgical removal of part of the elephantiasis tissue, this patient had lost more than 10 kg of weight of her left lower leg. The shape could be normalized. Redon drainage was removed 10 days later. A compression bandage was applied and DLT restarted. The leg became painless and mobility was markedly improved.

Two weeks later in ambulatory care, she presented a skin necrosis with lymphorrhea on the distal part of the suture. After necrectomy, vacuum-assisted closure was applied and within 3 weeks lymphorrhea stopped. Rapid granulation was achieved. Reepithelialization was completed within 5 weeks. The maximum diameter of the left lower leg had been reduced from 83 cm (before liposuction) to 53 cm (after debulking surgery) (Fig 5c, d).

## DISCUSSION

Lipo-lymphedema is a combination of lymphedema and lipedema. In early stages, DLT improves lymphatic flow and decreases pain in lipedema.^15^ Conservative treatment, however, does neither stop further growth of adipose tissue nor has it any impact on the disturbed adipose tissue turnover.^6^^,^^7^ Elephantiasis is an end-stage lymphatic disease, where DLT is necessary to control the disease but seldom results in significant improvement.^1^

Liposuction has been shown to improve lipedema with excellent long-term outcome.^8^^-^12 Microcannular liposuction with tumescent anesthesia leads to significant reduction of adipose tissue, reduction of pain, and bruising while preserving lymphatics.^1^ The same procedure may also improve lymphedema when combined with DLT—at least for the arms.^16^^-^18

In elephantiasis, however, liposuction is of limited value due to tissue fibrosis. In cases with severe impairment nonresponsive to conservative or minimally invasive surgical procedures, debulking surgery can be used to improve quality of life.^13^,^19^^-^22 The basic principle is the excision of affected cutaneous and subcutaneous tissue—dermato-fibro-lipectomy.

This procedure has to be used as an adjunct to DLT and should be considered for patients who fulfil the following criteria: end-stage chronic lymphedema accompanied by increased difficulty in providing effective DLT (highly disfigured extremity) and increased frequency of soft tissue infections.^1^

Histologic evaluation of removed subcutaneous tissue in our patients demonstrated phlebectomy, ectatic lymphatic vessels, and formation of large pseudocysts embedded in a fibrotic matrix. The slit-like and ectatic lymphatic vessels were podoplanin negative. Podoplanin is a marker of lymphatic endothelial cells. Recently, a subtype with low expression of podoplanin was identified—precollector lymphatic endothelium.^23^ In knockout mice models (podoplanin −/− or podoplanin +/−), large ectatic podoplanin-negative vessels, impaired lymphatic network patterning, and lymphedema were observed.^24^ These findings resemble what we have found in the 2 elephantiasis nostras patients. The absence of podoplanin would also explain the disturbed local immune defence, because podoplanin is involved in cell-cell interaction with immune cells.

Several approaches for debulking surgery have been developed. Excision of large amounts of subcutaneous tissue with insufficient lymphatics followed by adaption to muscle aponeurosis and/or muscular tissue after partial removal of aponeurosis have been performed for a long time. The idea behind this technique was de novo formation of lymphatics and blood vessels from sprouting of intramuscular blood and lymphatic vessels.^19^^-^22

However, compliance with maintenance of DLT postoperatively becomes a major critical issue for long-term success of excisional surgery. Without adequate postoperative DLT surgery fails.^1^

We observed delayed secondary wound healing and secondary wound infections. It has been demonstrated that ASA score is an independent predictor of postoperative wound infection.^25^ The present cases suggest that insufficient ambulatory compression therapy is another factor responsible for secondary ulcerations and delayed healing.

Ulcerations in lymph-edematous extremities possess a therapeutic challenge. The use of vacuum closure seems to be particularly valuable in such patients because it allows to treat not only the open wound, improve granulation, and microcirculation but to obstruct lymphorrhea.^21^^,^^26^^,^^27^

## CONCLUSIONS

Debulking surgery is a second-line treatment for patients with advanced lipo-lymphedema and elephantiasis nostras. Postoperative complications may occur, but they are manageable. Patients can realize a better quality of life in a situation that cannot be controlled by DLT anymore. Nevertheless, debulking surgery is an adjunctive treatment to DLT and should be performed only in specialized centers.

## References

[1] International Society of Lymphology (2013). The diagnosis and treatment of peripheral lymphedema: 2013 Consensus Document of the International Society of Lymphology. Lymphology.

[2] Kasseroller RG (1998). The Vodder School: the Vodder method. Cancer.

[3] Földi E (2012). Therapy of lymphedema. Hautarzt.

[4] Stout N, Partsch H, Szolnoky G (2012). Chronic edema of the lower extremities: international consensus recommendations for compression therapy clinical research trials. Int Angiol.

[5] Lee BB, Kim YW, Kim DI, Hwang JH, Laredo J, Neville R (2008). Supplemental surgical treatment to end stage (stage IV-V) of chronic lymphedema. Int Angiol.

[6] Meier-Vollrath I, Schmeller W (2004). Lipoedema—current status, new perspectives. J Dtsch Dermatol Ges.

[7] Suga H, Araki J, Aoi N, Kato H, Higashino T, Yoshimura K (2009). Adipose tissue remodeling in lipedema: adipocyte death and concurrent regeneration. J Cutan Pathol.

[8] Wollina U, Heinig B (2012). Tumescent microcannular (laser-assisted) liposuction in painful lipedema. Eur J Aesthet Med Dermatol.

[9] Peled AW, Slavin SA, Brorson H (2012). Long-term outcome after surgical treatment of lipedema. Ann Plast Surg.

[10] Rapprich S, Dingler A, Podda M (2011). Liposuction is an effective treatment for lipedema: results of a study with 25 patients. J Dtsch Dermatol Ges.

[11] Wollina U, Goldman A, Heinig B (2010). Microcannular tumescent liposuction in advanced lipedema and Dercum's disease. G Ital Dermatol Venereol.

[12] Schmeller W, Meier-Vollrath I (2006). Tumescent liposuction: a new and successful therapy for lipedema. J Cutan Med Surg.

[13] Kim DI, Lee BB, Huh S, Lee SJ, Hwang JH, Kim YI (1998). Excision of subcutaneous tissue and deep muscle fascia for advanced lymphedema. Lymphology.

[14] Daabiss M (2011). American Society of Anaesthesiologists physical status classification. Indian J Anaesthesia.

[15] Szolnoky G, Varga E, Varga M, Tuczai M, Dósa-Rácz E, Kemény L (2011). Lymphedema treatment decreases pain intensity in lipedema. Lymphology.

[16] Brorson H (2012). Liposuction normalizes—in contrast to other therapies—lymphedema-induced adipose tissue hypertrophy. Handchir Mikrochir Plast Chir.

[17] Brorson H, Svensson H, Norrgren K, Thorsson O (1998). Liposuction reduces arm lymphedema without significantly altering the already impaired lymph transport. Lymphology.

[18] Sistrunk WE (1998). Liposuction combined with controlled compression therapy reduces arm lymphedema more effectively than controlled compression therapy alone. Plast Reconstr Surg.

[19] Auchincloss H (1930). New operation for elephantiasis. Puerto Rico J Publ Health Trop Med.

[20] Homans J (1936). The treatment of elephantiasis of the legs. A preliminary report. N Engl J Med.

[21] Heinig B, Wollina U (2003). Surgery in congenital lymphedema: a follow-up of 50 years. Int J Low Extrem Wounds.

[22] Wang HD, Shridharani SM, Tufaro AP (2013). Lymphedema. Eplasty.

[23] Lee S, Choi I, Hong YK (2010). Heterogeneity and plasticity of lymphatic endothelial cells. Semin Thromb Hemost.

[24] Schacht V, Ramirez MI, Hong YK (2003). T1alpha/podoplanin deficiency disrupts normal lymphatic vasculature formation and causes lymphedema. EMBO J.

[25] Woodfield JC, Beshay NM, Pettigrew RA, Plank LD, van Rij AM (2007). American Society of Anesthesiologists classification of physical status as a predictor of wound infection. ANZ J Surg.

[26] Wollina U, Hansel G, Krönert C, Heinig B (2010). Using VAC to facilitate healing of traumatic wounds in patients with chronic lymphoedema. J Wound Care.

[27] Tauber R, Schmid S, Horn T (2013). Inguinal lymph node dissection: epidermal vacuum therapy for prevention of wound complications. J Plast Reconstr Aesthet Surg.

